# The Pro- and Anti-Inflammatory Cytokine Profile in Keratoconus as a Predictor of Five-Year Corneal Cross-Linking Outcomes

**DOI:** 10.3390/ijms27093768

**Published:** 2026-04-23

**Authors:** Tiana Petrovic, Svetlana Stanojlovic, Sanja Petrovic Pajic, Borivoje Savic, Tanja Kalezic, Nada Avram, Vesna Sobot, Marko Svetel, Milica Jeremic Kaplarevic, Vladimir Perovic

**Affiliations:** 1 Clinic for Eye Diseases, University Clinical Center of Serbia, 11000 Belgrade, Serbia; tianapetrovic93@gmail.com (T.P.);; 2 Faculty of Medicine, University of Belgrade, 11000 Belgrade, Serbia; 3 Ophthalmology Department, University Hospital Foča, 73300 Foča, Bosnia and Herzegovina; 4 Institute of Microbiology and Immunology, Faculty of Medicine, University of Belgrade, 11000 Belgrade, Serbia

**Keywords:** keratoconus, cytokine, tears, CXL

## Abstract

The aim of this study was to evaluate tear cytokine and chemokine profiles in keratoconus (KC) and to assess their association with long-term tomographic outcomes after corneal collagen cross-linking (CXL). In this cross-sectional observational study, 30 KC eyes and nine healthy controls were enrolled. KC severity was graded using the modified Amsler–Krumeich classification. Tear samples were collected and analyzed using multiplex bead-based immunoassays (LEGENDplex™) for cytokines, chemokines, and TGF-β1. Patients were followed for five years after CXL. Treatment response was categorized according to corneal flattening (<1 D, 1–3 D, >3 D). Individual cytokine levels showed no major differences between the KC and controls, although IL-6 and MCP-1 tended to be higher in the KC group. However, IP 10 and IL-17 were higher in controls (*p* < 0.05). In contrast, multiple pro-/anti-inflammatory ratios (TNF/TGF-β1, IL-17/IL-10, MCP-1/IL-10, IL-6/IL-10, IL-8/TGF-β1, MCP-1/TGF-β1, IL-6/TGF-β1, and IL-8/IL-10) were significantly elevated in the KC group (*p* < 0.05), indicating immune imbalance. After five years, all treated eyes remained stable or flattened. Lower baseline MCP-1 and IL-8 levels correlated with greater postoperative corneal flattening (*p* < 0.05). Keratoconus is characterized by disturbed tear immune homeostasis rather than isolated cytokine elevation. Lower preoperative inflammatory activity may predict a more favorable biomechanical response to CXL, supporting the potential role of tear cytokine profiling in patient stratification and prognostication.

## 1. Introduction

Keratoconus (KC) is a progressive, typically bilateral but asymmetric corneal ectasia, characterized by stromal thinning, irregular astigmatism, and progressive visual loss, which often leads to a substantial reduction in quality of life and, in advanced cases, to the need for keratoplasty [1]. Although KC was long considered a non-inflammatory degenerative disorder, evidence from tear film and corneal tissue studies has shifted this view toward chronic, low-grade inflammatory or para-inflammatory activation at the ocular surface [2,3,4]. An increased inflammatory response has been consistently demonstrated in the KC tear film compared with healthy controls [3,5].

Classic studies have shown elevated IL-6 and TNF-α levels, as well as increased MMP-9 activity, in both subclinical and manifest KC, indicating early inflammatory involvement [6,7]. Elevated levels of IL-1β, IL-6, TNF-α, and IFN-γ, together with the dysregulation of anti-inflammatory mediators (e.g., IL-10), support a sustained imbalance between effector and immunoregulatory pathways [6,7,8]. However, immunological heterogeneity exists, with some authors describing KC cases with lower cytokine concentrations [2]. Activation of oxidative stress pathways in the KC epithelium suggests an interaction between inflammation, redox imbalance, and stromal biomechanical weakening [9]. Increased densities of antigen-presenting cells and T-cell activation on the KC ocular surface further support the involvement of cellular immunity [10].

Higher tear levels—and particularly higher ratios—of IL-6, IL-8, or TNF-α relative to IL-10 correlate with steeper keratometry and reduced minimal stromal thickness [7]. Clinical factors such as atopy and chronic eye rubbing are also associated with increased tear inflammation, suggesting a synergistic effect between mechanical and immune stressors in genetically predisposed corneas [5,11].

Corneal collagen cross-linking (CXL) remains the standard of care for halting ectatic progression by increasing stromal stiffness and enzymatic resistance [12,13]. However, CXL induces keratocyte apoptosis and reactive oxygen species generation, thereby modulating postoperative ocular surface immunity [8,14,15]. Several studies have evaluated whether tear film cytokine changes after CXL influence corneal remodeling outcomes [12,13,16]. Chemokines such as IL-8 and MCP-1 promote leukocyte recruitment and epithelial stress and are associated with more advanced disease [5,7]. Lower pre-CXL IL-6 or MCP-1 levels are correlated with greater long-term corneal flattening and improved stability, suggesting that a less inflamed baseline state may favor a more efficient biomechanical response [12,13,16].

KC represents a chronic, immune-modulated ectatic disorder driven by dysregulated cytokine and chemokine signaling at the ocular surface [2,3,4,7,8]. Understanding these inflammatory profiles may enhance biomarker-based patient stratification and improve prognostication in progressive disease treated with CXL [12,13,16].

## 2. Results

### 2.1. Study Population Characteristics, Disease Severity and Corneal Cross-Linking Outcomes

A total of 30 eyes with keratoconus and nine healthy control eyes were included in the multiplex cytokine and chemokine analysis of tear fluid. All KC eyes underwent corneal collagen cross-linking (CXL) and were examined at 1, 3, and 6 months, and at 1, 3, and 5 years after treatment. Corneal tomography was performed at each visit to evaluate structural changes and disease stability. Based on tomographic parameters, eyes were classified into four grades of severity: 12 were Grade 1 (40%), 6 were Grade 2 (20%), 7 were Grade 3 (23.3%), and 4 were Grade 4 (13.3%). One eye was excluded from the analysis because of an invalid tomographic scan (Table 1).

After five years of follow-up, all treated corneas remained stable or showed measurable flattening, confirming the long-term efficacy of CXL in preventing KC progression. According to tomographic changes along the tangential axis (K-max, K-mean, and minimal corneal thickness, MCT), the outcomes were grouped as follows: (1) stable parameters or <1 D corneal flattening in 13 eyes (43.3%); (2) 1–3 D flattening in 7 eyes (23.3%); and (3) >3 D flattening in 9 eyes (30%). No cases of postoperative steepening were observed.

### 2.2. Cytokine Levels

**Figure 1:**
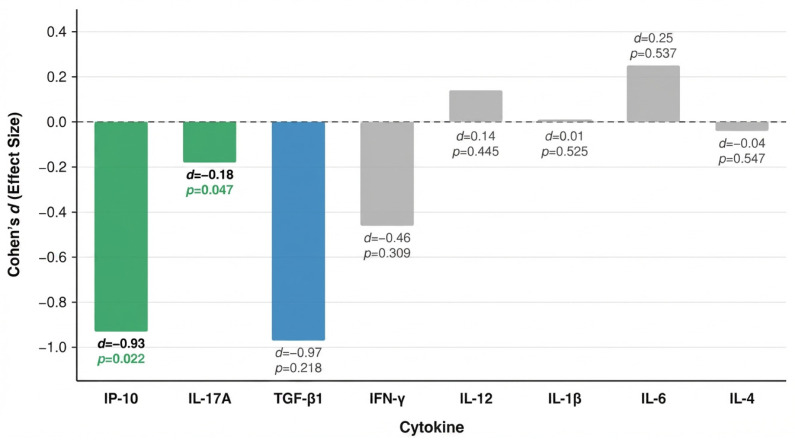
Effect sizes (Cohen’s d) for individual tear cytokine comparisons between keratoconus patients and healthy controls. Negative values indicate lower concentrations in patients. Green bars denote statistical significance (*p* < 0.05); blue bars denote large effect size (|d| ≥ 0.8) without reaching significance; and gray bars denote small, non-significant differences.

**Figure 2:**
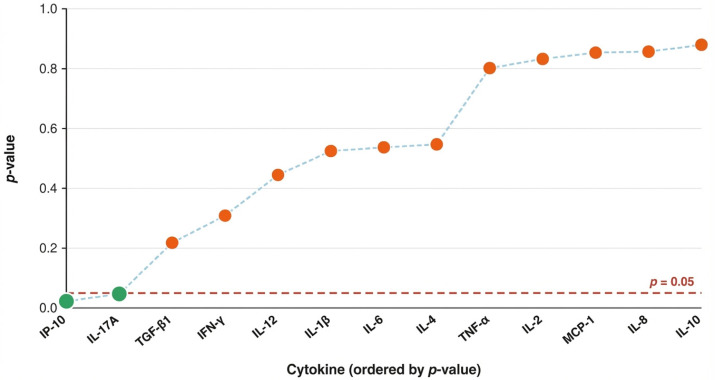
Individual tear cytokine *p*-values (Mann–Whitney U) for the comparison between keratoconus patients and healthy controls, ranked from most to least significant. Green dots indicate *p* < 0.05 and orange dots indicate *p* ≥ 0.05. The dashed red line marks the significance threshold.

The tear fluid levels of cytokines (IL-1β, IL-2, IL-6, IL-10, IL-12p70, IL-17A, TNF-α, and IFN-γ), chemokines (MCP-1/CCL2, IL-8/CXCL8, and IP-10/CXCL10), and growth factor TGF-β1 were analyzed in KC and control groups. Mean IL-6 and MCP-1 concentrations were higher in the KC group, although the differences were not statistically significant (*p* > 0.05). In contrast, both IP-10 and IL-17A levels were higher in the control group and reached statistical significance (*p* < 0.05) (Figure 1 and Figure 2). The remaining cytokines showed only minor, non-significant variations between the groups, with a similar degree of fluctuation in both KC and control tears.

### 2.3. Correlations Between Pro-Inflammatory and Anti-Inflammatory Mediators

**Figure 3:**
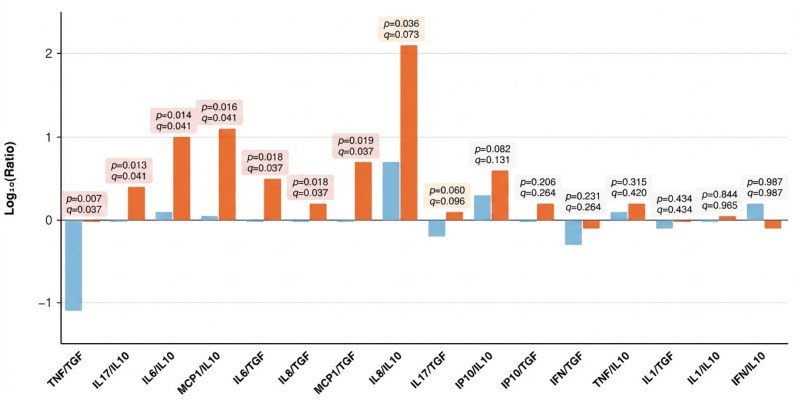
Median log-transformed pro-/anti-inflammatory cytokine ratios in controls (blue) and keratoconus (red) patients among subjects with jointly detectable cytokines. The ratios are ordered by permutation-calibrated *p*-value. The annotations show permutation *p*-values and Benjamini–Hochberg FDR-corrected q-values. Red annotation text: q < 0.05; orange: q < 0.10; gray: not significant.

**Figure 4:**
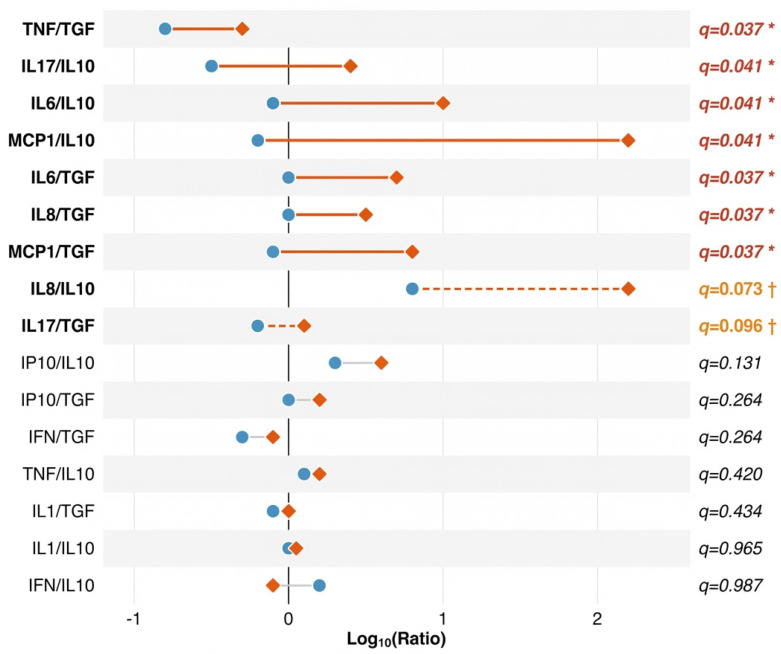
Dumbbell plot showing the shift in median log-transformed cytokine ratios from controls (blue circles) to keratoconus patients (red diamonds) among subjects with jointly detectable cytokines. The solid connecting lines indicate FDR-corrected significance at q < 0.05 marked with *; dashed lines indicate q < 0.10 marked with †; and gray lines indicate non-significant differences. FDR-corrected q-values are shown on the right (q < 0.05; q < 0.10).

**Figure 5:**
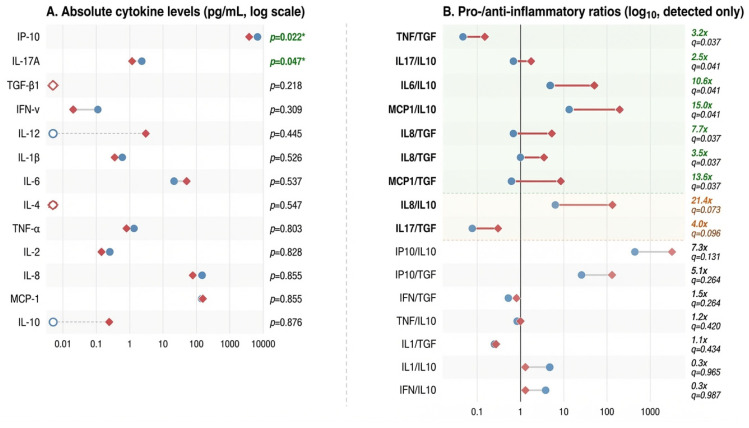
Side-by-side comparison of individual cytokine concentrations and pro-/anti-inflammatory cytokine ratios in keratoconus patients versus healthy controls. (**A**) Absolute tear cytokine levels (pg/mL, log scale). Blue circles represent median control values; red diamonds represent median patient values. Only IP-10 and IL-17A differed significantly between the groups (*p* < 0.05, Mann–Whitney U marked with *). (**B**) Pro-/anti-inflammatory cytokine ratios among subjects with jointly detectable cytokines ordered by permutation-calibrated significance. Fold change and FDR-corrected q-values are shown on the right. Seven ratios reached significance at q < 0.05 (green) and two were suggestive at q < 0.10 (orange), all featuring pro-inflammatory numerators (TNF-α, IL-6, IL-8, IL-17A, and MCP-1) over anti-inflammatory denominators (TGF-β1 and IL-10).

To better characterize the inflammatory balance, correlations between pro-inflammatory and anti-inflammatory mediators were examined. Statistically significant differences were found in the distribution of the following ratios between the KC and control groups: TNF/TGF-β1, IL-17/IL-10, MCP-1/IL-10, IL-6/IL-10, IL-8/TGF-β1, MCP-1/TGF-β1, IL-6/TGF-β1, and IL-8/IL-10 (*p* < 0.05) (Figure 3, Figure 4 and Figure 5). These patterns reflect a clear disturbance in the balance between pro- and anti-inflammatory activity in the KC tear film.

### 2.4. Cytokine Level Correlation to Keratoconus Grade and Corneal Cross-Linking Outcomes

**Scheme 1:**
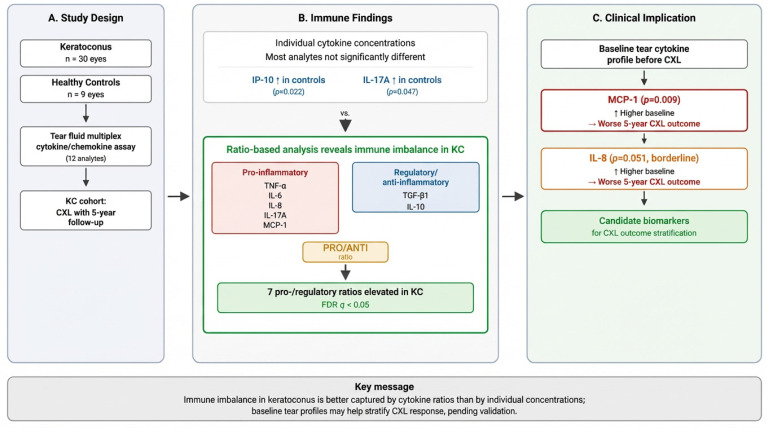
Schematic representation of the study design and the relationship between pro- and anti-inflammatory factors on the ocular surface as predictive biomarkers for the development of keratoconus, as well as IL-8 and MCP-1 as predictive biomarkers of long-term corneal cross-linking (CXL) outcomes.

**Figure 6:**
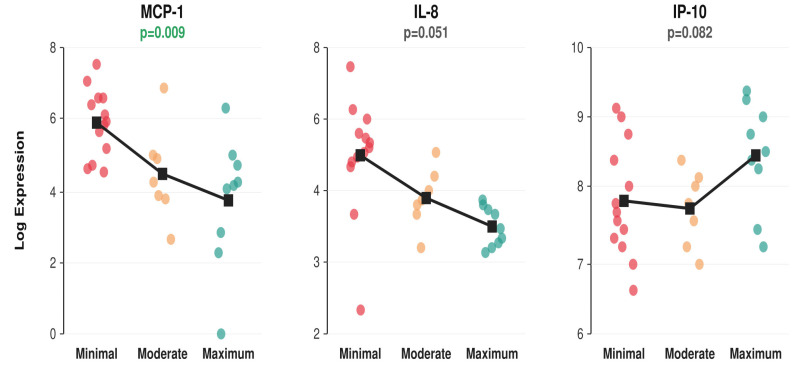
Baseline concentrations (log-transformed) of MCP-1, IL-8, and IP-10 across corneal flattening response groups five years after CXL. Red: minimal response (<1 D); orange: moderate (1–3 D); green: maximum (>3 D). Black squares and connecting lines represent the group means. *p*-values are from the Kruskal–Wallis test.

**Figure 7:**
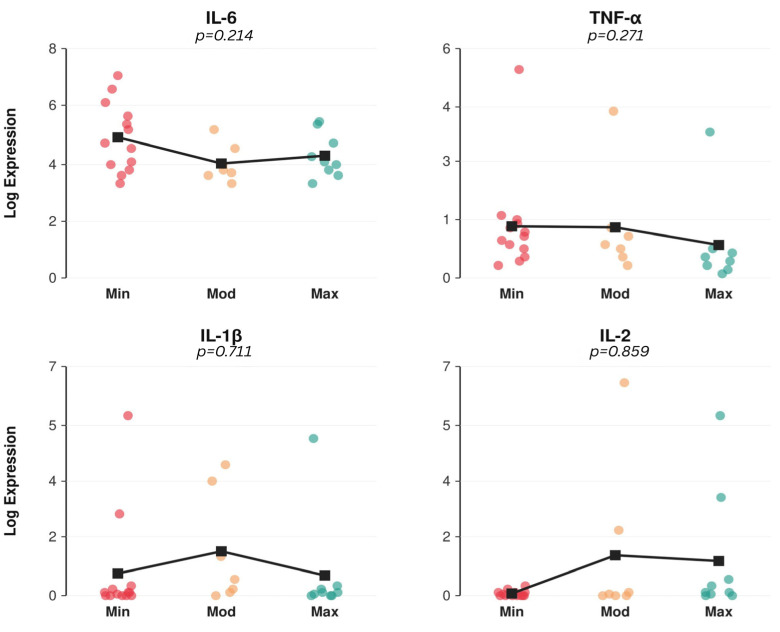
Red Baseline concentrations (log-transformed) of IL-6, TNF-α, IL-1β, and IL-2 across corneal flattening response groups. The layout and color coding is as in Figure 6. None reached statistical significance.

**Figure 8:**
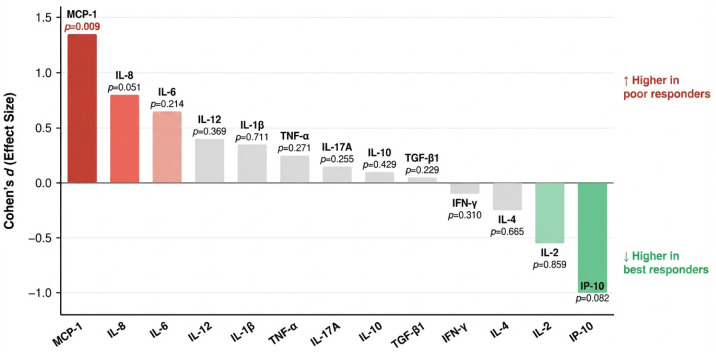
Waterfall plot ranking all the cytokines by absolute effect size (Cohen’s d) for the association between baseline concentration and treatment response. Positive values (red) indicate higher expression in patients with minimal flattening; negative values (green) indicate higher expression in patients with maximum flattening. The color intensity reflects the statistical significance.

When cytokine levels were compared across keratoconus grades, no statistically significant differences were detected. However, a negative correlation was observed between baseline MCP-1 and IL-8 concentrations and the degree of corneal flattening measured five years after CXL. Eyes with lower pre-operative MCP-1 and IL-8 levels achieved greater postoperative flattening, suggesting that reduced baseline inflammatory activity may predict a more favorable biomechanical response to treatment (Scheme 1 and Figure 6). Other cytokines or their ratios did not show any statistically important correlation to outcome (Figure 7 and Figure 8).

In summary, although individual cytokine levels did not differ markedly between the KC group and the controls, the increased ratios of inflammatory mediators point to an imbalance in tear immunoregulation. Moreover, the association between lower pre-CXL cytokine levels and better long-term corneal flattening supports the potential of tear cytokine profiling as a predictive biomarker of therapeutic outcome in keratoconus.

## 3. Discussion

Our findings reinforce the view that keratoconus (KC) represents a chronic, immune-mediated corneal disorder in which the relative balance between inflammatory mediators—rather than their absolute concentrations—plays a decisive role in disease behavior [17,18,19,20]. Meta-analytic evidence confirms that IL-1β, IL-6, IL-8, TNF-α and related cytokines are frequently elevated in KC and are associated with steeper corneas, reduced stromal thickness, epithelial instability and increased ocular-surface reactivity [15,18,19,20]. These mediators contribute to stromal degradation through the activation of MAPK and NF-κB signaling pathways and the stimulation of matrix metalloproteinases, including MMP-9 [20].

Proteomic and metabolomic analyses corroborate these mechanisms, demonstrating increased expression of inflammatory, oxidative-stress and extracellular matrix-remodeling proteins, along with the accumulation of oxidative metabolites and signatures of lipid peroxidation in KC [21,22,23,24]. Altered cytokine ratios, such as IL-6/IL-10 and IL-17/TGF-β1, further indicate insufficient anti-inflammatory counter-regulation and dysregulated immune homeostasis [18,20]. Together, these findings support oxidative imbalance as a central driver of keratocyte apoptosis, MMP activation and stromal thinning.

Variability in the specific mediators highlights the biological heterogeneity of KC. IL-17A levels correlate with disease severity and may reflect Th17-driven inflammation. IP-10 (CXCL10) participates in corneal epithelial remodeling and wound-healing dynamics, with higher or fluctuating levels potentially reflecting physiological homeostatic signaling rather than the absence of inflammation [18,25].

Lower baseline MCP-1 and IL-8 may favor better corneal flattening after CXL because they likely reflect a less inflamed and less proteolytically active corneal microenvironment, in which the biomechanical effect of CXL can be more effectively translated into topographic improvement. IL-8, as a key neutrophil chemoattractant, is associated with active inflammatory stress and tissue damage, whereas MCP-1 (CCL2) is linked to monocyte/macrophage-driven remodeling and extracellular matrix turnover; therefore, higher levels of these mediators may indicate ongoing stromal instability that can attenuate the flattening response [12,26]. This interpretation is supported by evidence that better CXL outcomes correlate with lower proteolytic activity and a more favorable molecular profile of the cornea [27]. Importantly, keratoconus is biologically heterogeneous, with molecular alterations present even outside the cone region, which may explain why eyes with similar tomographic parameters can show different responses to treatment [28]. This heterogeneity suggests that inflammatory status may vary between apparently comparable cases and may contribute to differences in biomechanical responsiveness after CXL. In this context, baseline inflammatory markers, such as IL-8 and possibly MCP-1, may help identify a “low-inflammatory” phenotype more likely to achieve meaningful flattening, whereas eyes with a higher inflammatory burden may represent a subgroup with a less favorable response profile. Thus, these findings support the concept of biomarker-based clinical stratification as a potential adjunct to tomographic and clinical assessment [12,29]. However, given the limited sample size, these associations should be interpreted with caution and require validation in larger cohorts.

Several clinical studies have shown that elevated pre-CXL levels of IL-6, IL-8, TNF-α and related mediators are associated with weaker postoperative flattening or residual tomographic instability [7,17,22], although further research is needed to evaluate their long-term impact. In parallel, matrix metalloproteinases, particularly MMP-2 and MMP-9, represent key downstream effectors contributing to stromal thinning and ectasia progression [30].

Collectively, these data support a unified model in which KC progression is maintained by a sustained imbalance between pro-oxidative, proteolytic and immune-regulatory mechanisms [20,22,23,24]. From a translational perspective, baseline cytokine and chemokine ratios may help stratify patients by expected response to CXL and identify those who could benefit from pre-treatment optimization of the ocular surface, including management of allergy, dry eye or meibomian gland dysfunction [17,20,22]. Incorporating such immune profiling into a multimodal framework that also includes tomography, epithelial thickness mapping and biomechanical evaluation may improve risk stratification and long-term prognostication in KC [20,22,23].

Nevertheless, several limitations of this study should be acknowledged. Tear sampling using Schirmer strips may introduce variability related to reflex tearing, strip contact time and elution efficiency. Although all samples were collected under a standardized protocol (centrifugation at 13,000 rpm for 15 min at 4 °C with a fixed elution volume of 300 μL), this source of variation cannot be fully eliminated and may limit direct comparison between absolute cytokine concentrations across studies. In addition, the cohort was derived from a single tertiary center and consisted predominantly of young male patients, which may limit generalizability, as demographic, environmental and lifestyle factors may influence baseline tear cytokine profiles.

## 4. Materials and Methods

### 4.1. Study Design

This study was designed as a cross-sectional and observational study for cytokine analysis but was longitudinal for outcome. This study was conducted in full compliance with institutional guidelines and the Declaration of Helsinki, and was approved by the Ethics Committee of the Clinical Center of Serbia. Written informed consent was obtained from all participants prior to enrollment. The diagnosis of keratoconus relied on corneal tomography (Orbscan II, Bausch & Lomb, Rochester, NY, USA) at baseline and at every follow-up visit. Disease severity was graded using the modified Amsler–Krumeich classification, which integrates slit-lamp biomicroscopic findings, mean central keratometry, changes in spherical and cylindrical refraction, and corneal thickness. A structured history captured eye rubbing as well as ocular and systemic allergy.

For the cytokine outcome analysis, assuming an expected correlation coefficient of r = 0.5 (moderate effect size), a minimum of 29 observations was required to achieve 80% statistical power at α = 0.05 (two-tailed). The cohort comprised 27 KC subjects (30 eyes; age 30.03 ± 6.5 years; male/female = 23/3) and 9 healthy controls (9 eyes; age 29.8 ± 3.8 years; male/female = 7/2). Controls were proportionally matched to patients by age and sex. None had known ophthalmological disease, prior ocular surgery or active ocular surface disease. One of the originally enrolled 10 controls was excluded after being diagnosed with a dust-mite allergy, due to the potential confounding effect of atopic sensitization on tear cytokine levels.

By KC grade, eyes were distributed as Grade 1 (12), Grade 2 (6), Grade 3 (7), and Grade 4 (4) (Table 1). Advancing grades were accompanied by a grade-dependent increases in keratometric indices (K1, K2, K-mean, and K-max) and a reduction in pachymetric indices (central corneal thickness—CCT; thinnest corneal thickness—TCT), alongside characteristic clinical signs, notably Fleischer ring and Vogt striae.

Post-treatment monitoring after corneal cross-linking was scheduled at 1, 3, and 6 months, and then at 1, 3, and 5 years. Corneal tomography was obtained at each timepoint. Tomographic change along the tangential axis served to classify outcomes into three categories: (minimal) stable parameters or <1 D corneal flattening, (moderate) 1–3 D flattening, and (maximal) >3 D flattening. No patient exhibited corneal steepening.

### 4.2. Tear Sampling

For tear sampling, sterile Schirmer strips were applied and immediately placed into sterile perforated microcentrifuge tubes nested within larger collection tubes, followed by centrifugation at 13,000 rpm for 15 min at 4 °C. The eluted tear fluid (300 μL) was transferred to fresh sterile microtubes and used for downstream analyses. Tear sampling was performed before CXL treatment.

The quantification of soluble tear factors included cytokines (IL-1β, IL-2, IL-6, IL-10, IL-12p70, IL-17A, TNF-α, and IFN-γ), chemokines (MCP-1/CCL2, IL-8/CXCL8, and IP-10/CXCL10), and the growth factor TGF-β1, measured with LEGENDplex™ (BioLegend Inc., San Diego, CA, USA), according to the manufacturer’s instructions. Bead acquisition and signal capture for both Cytometric Bead Array and LEGENDplex™ assays were performed on a BD FACS Canto™ II flow cytometer using BD FACSDiva software Diva v6.0 (BD Biosciences, San Jose, CA, USA). Absolute analyte concentrations were derived using FCAP Array v3.0 (BD Biosciences, USA) for Cytometric Bead Array data and the LEGENDplex™ Data Analysis Software Suite, Version 8.0 (BioLegend Inc., San Diego, CA, USA) for LEGENDplex™ data, based on analyte-specific standard curves.

### 4.3. Statistical Analysis

Data are presented as medians with interquartile ranges (IQR) for continuous variables and as detection frequencies (the number and percentage above the assay limit of detection) for each cytokine. Comparisons of cytokine levels between the groups (keratoconus patients versus healthy controls) were conducted using two-sided Mann–Whitney U tests. Below-detection-limit (BDL) values, which are reported as zeros, were kept as tied lowest ranks for the rank-based tests. For cytokine ratio analyses, a two-part hurdle framework was employed to address BDL-driven selection bias: (i) Fisher’s exact test compared joint detectability of the numerator and denominator cytokines between the groups, and (ii) one-sided Mann–Whitney U tests compared ratio magnitudes among the subjects with both cytokines detectable. The two components were combined using Fisher’s method (X^2^ = −2[ln(p_1_) + ln(p_2_)]) and were calibrated empirically by the permutation of case/control labels (10,000 iterations). Ratios requiring fewer than 4 detectable subjects per group were excluded from inferential testing. Associations between cytokine levels and ordinal disease stage at treatment were assessed using permutation-calibrated Spearman rank correlation and the Jonckheere–Terpstra trend test, which exploit the ordered nature of disease stages more efficiently than Kruskal–Wallis for monotonic alternatives. All *p*-values were adjusted for multiple comparisons using the Benjamini–Hochberg false discovery rate (FDR) procedure. FDR was applied per-family for ratio analyses (within each anti-inflammatory denominator family) and across all cytokines for individual comparisons and stage-association tests. Significance was declared at *p* < 0.05; results at 0.05 ≤ *p* < 0.10 were reported as suggestive. Effect sizes are reported as Spearman’s ρ (trend tests), rank-biserial correlation (Mann–Whitney), and median fold-changes (ratios). All analyses were performed in Python 3.11 using SciPy, statsmodels, and custom permutation routines.

## 5. Conclusions

In this long-term cohort of keratoconus eyes treated with standard epithelium-off CXL, individual tear cytokine levels did not differ markedly from healthy controls; however, multiple ratios of pro- to anti-inflammatory mediators were significantly altered, indicating a disturbed immune balance at the ocular surface. Lower pre-operative IL-8 and MCP-1 concentrations were associated with greater corneal flattening five years after CXL, suggesting that a less inflamed tear microenvironment may favor more effective stromal remodeling. These results support a shift from single-marker interpretation toward ratio-based, multiplex tear-film profiling and indicate that baseline cytokine patterns could contribute to risk stratification and prognostication in KC. Integrating tear immune signatures with tomographic and epithelial metrics may ultimately enable more personalized decisions about the timing of CXL, the need for retreatment and the potential role of adjunctive anti-inflammatory therapy.

## Figures and Tables

**Table 1:** Patient characteristics, including gender, affected eye, keratoconus stage (Amsler–Krumeich), and medical history.

Patient Characteristics	N (%)
Gender	
Male	23 (88.5%)
Female	3 (11.5%)
Eye	
Left	17 (57%)
Right	13 (43%)
Keratoconus grade (Amsler–Krumeich)	
I	12 (41%)
II	6 (21%)
III	7 (24%)
IV	4 (14%)
Family history of keratoconus	0
Eye rubbing	7 (27%)
Use of contact lenses	7 (27%)
Allergies	9 (31%)

## Data Availability

The original contributions presented in this study are included in the article. Further inquiries can be directed to the corresponding author.

## Abbreviations

The following abbreviations are used in this manuscript:

KC	Keratoconus
CXL	Corneal cross-linking
TCT	Thinnest corneal thickness
CCT	Central corneal thickness
